# Deciphering Respiratory-Virus-Associated Interferon Signaling in COPD Airway Epithelium

**DOI:** 10.3390/medicina58010121

**Published:** 2022-01-13

**Authors:** Hong Guo-Parke, Dermot Linden, Sinéad Weldon, Joseph C. Kidney, Clifford C. Taggart

**Affiliations:** 1 Wellcome Wolfson Institute for Experimental Medicine, School of Medicine, Dentistry & Biomedical Sciences, Queens University Belfast, Belfast BT9 7AE, UK; h.guoparke@qub.ac.uk (H.G.-P.); D.Linden@qub.ac.uk (D.L.); s.weldon@qub.ac.uk (S.W.); 2 Department of Respiratory Medicine, Mater Hospital Belfast, Belfast BT14 6AB, UK; joe.kidney@belfasttrust.hscni.net

**Keywords:** COPD, airway epithelial cells, interferon signaling, respiratory virus, viral infection

## Abstract

COPD is a chronic lung disorder characterized by a progressive and irreversible airflow obstruction, and persistent pulmonary inflammation. It has become a global epidemic affecting 10% of the population, and is the third leading cause of death worldwide. Respiratory viruses are a primary cause of COPD exacerbations, often leading to secondary bacterial infections in the lower respiratory tract. COPD patients are more susceptible to viral infections and associated severe disease, leading to accelerated lung function deterioration, hospitalization, and an increased risk of mortality. The airway epithelium plays an essential role in maintaining immune homeostasis, and orchestrates the innate and adaptive responses of the lung against inhaled and pathogen insults. A healthy airway epithelium acts as the first line of host defense by maintaining barrier integrity and the mucociliary escalator, secreting an array of inflammatory mediators, and initiating an antiviral state through the interferon (IFN) response. The airway epithelium is a major site of viral infection, and the interaction between respiratory viruses and airway epithelial cells activates host defense mechanisms, resulting in rapid virus clearance. As such, the production of IFNs and the activation of IFN signaling cascades directly contributes to host defense against viral infections and subsequent innate and adaptive immunity. However, the COPD airway epithelium exhibits an altered antiviral response, leading to enhanced susceptibility to severe disease and impaired IFN signaling. Despite decades of research, there is no effective antiviral therapy for COPD patients. Herein, we review current insights into understanding the mechanisms of viral evasion and host IFN antiviral defense signaling impairment in COPD airway epithelium. Understanding how antiviral mechanisms operate in COPD exacerbations will facilitate the discovery of potential therapeutic interventions to reduce COPD hospitalization and disease severity.

## 1. Introduction

Chronic obstructive pulmonary disease (COPD) is a common chronic inflammatory airways disease affecting 1.2 million people in the UK, and 10% of the global population aged over 45 [1]. It is characterized by progressive and irreversible airflow obstruction, and chronic persistent pulmonary inflammation [1,2,3]. Respiratory virus infections are major drivers of acute COPD exacerbations, and are associated with COPD hospitalization, morbidity, and mortality [2,3]. In fact, COPD is the third leading cause of death globally, imposing a substantial social and economic burden [1,2,3].

The airway epithelium plays an essential role in maintaining immune homeostasis, and modulating the innate and adaptive immune response in the lung against inhaled and pathogen insults [4]. As a primary site of respiratory viral infection, airway epithelial cells act as the first line of host defense by maintaining barrier integrity and the mucociliary escalator to prevent virus binding and entry into the host [3,4]. It also initiates the innate and adaptive immune response by secreting an array of inflammatory mediators, such as cytokines, chemokines, growth factors, lipid mediators, inflammasomes, and proteases [3,5,6]. Upon viral invasion, airway epithelial cells rapidly recognize pathogens through pattern recognition receptors (PRRs) and intracellular viral sensors, and induce an antiviral state through the production of interferons (IFNs) [3,4,6]. Consequently, IFNs activate a downstream signaling cascade through the expression of various interferon-stimulated genes (ISGs) [2,3,4].

Substantial evidence suggests that the COPD airway epithelium is more susceptible to viral infection and associated severe diseases, leading to accelerated lung function deterioration, hospitalization, and an increased risk of mortality [2,3,7]. The Global Initiative for Chronic Obstructive Lung Disease (GOLD) acknowledges that COPD patients are among the worst affected by COVID-19, also known as severe acute respiratory syndrome coronavirus 2 (SARS-CoV-2) [8]. In addition, impaired antiviral immunity has been implicated in COPD airway epithelial cells in response to a range of respiratory viruses, including SARS-CoV-2 (severe acute respiratory syndrome coronavirus 2) [9,10,11,12,13]. However, much remains unknown about how respiratory viruses subvert or suppress the host antiviral response, leading to increased virulence of infection, and susceptibility to severe disease in COPD airways.

In this review, we initially discuss what is known about IFN antiviral defense signaling in airway epithelium, and COPD-associated impairment of IFN signaling. We also highlight current advances in understanding mechanisms of how respiratory viruses modulate or evade these antiviral defenses.

## 2. Virus Sensing Pathways in Airway Epithelium

**Figure 1:**
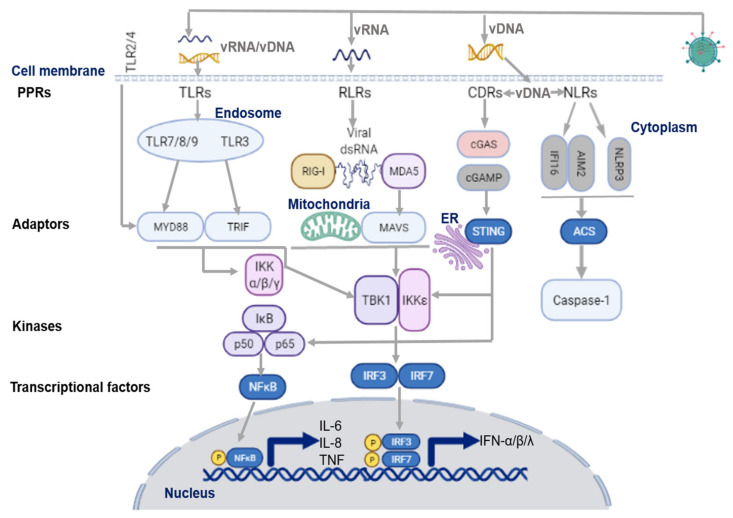
Virus sensing pathways on airway epithelium. TLR7/8/9 and TLR3 in the endosome sense ssRNA, and dsRNA of the viral genome, respectively. They then activate their adaptor proteins, MyD88 for the former, and TRIF for the latter. The cell surface receptors, TLR2/4/6, also recognize respiratory viruses in the airway by modulating the adaptor protein, MyD88, to induce the antiviral response. DNA and RNA viruses release their genomes in the cytoplasm, where host innate sensors for viral RNA/DNA reside. Upon ss/dsRNA binding, RLRs interact with the adaptor protein, MAVS, on the mitochondrial outer membrane. CDRs (such as cGAS receptor) sense dsDNA and the RNA: DNA hybrids, and induce the synthesis of cGAMP, which then binds to the adaptor protein, STING. NLRs recognize DNA and RNA viruses via the NLRP3 inflammasome. NLRP3 activates and recruits ASC and procaspase-1 to form an inflammasome complex; IFI16 can recruit STING in response to cytoplasmic DNA through a molecular mechanism yet to be described. NLRs modulate the recruitment of their adaptor protein, ASC, to induce inflammation through the activation and secretion of pro-IL-1β and pro-IL-18 via caspase-1. The maturation of these cytokines further stimulates the production of IFNs and other cytokines. On the other hand, adaptor proteins, MyD88, STING, and MAVS, stimulate downstream signaling cascades that involve multiple kinases (TBK1, IKKs), and finally lead to IRF3/7 phosphorylation and nuclear translocation. The primary consequence of these virus-sensing pathways is the induction of type I/III IFN and pro-inflammatory cytokines and chemokines. ASC, adapter protein apoptosis-associated speck-like protein containing a caspase recruitment domain; CDR, cytosolic DNA receptors; cGAS, cyclic GMP-AMP synthase; cGAMP, 2′3′guanosine-adenosine monophosphate; IFI16, interferon-g inducible protein 16; IFN, interferon; IKK, IκB kinase; IRF3, interferon regulatory factor 3; MAVS, mitochondrial antiviral-signaling protein; MyD88, myeloid differentiation primary response 88; NLR, (NOD)-like receptor; NLRP3, NOD-, LRR- and pyrin domain-containing protein 3; RIG-I, retinoic acid inducible gene-I; RLR, RIG-1-like receptors; ss/dsRNA, single-stranded/double-stranded RNA; vRNA/DNA, viral RNA/DNA; STING, stimulator of interferon genes; TANK, TRAF-associated NF-κB activator; TBK1, TANK binding kinase 1; TLR, toll like receptor; TRIF, toll/IL-1R domain-containing adaptor-inducing IFN-β. Figure created with BioRender.com (accessed on 10 October 2021).

The first phase of the antiviral response depends on the ability of host cells to rapidly sense the invasion of foreign pathogens, and promote the production of innate pro-inflammatory mediators, including IFNs, to impede virus-associated disease. Interaction with the airway epithelium represents the first encounter of virus with the human host, which initiates the innate immune response [3,4,7]. As illustrated in Figure 1, upon virus entry, airway epithelial cells rapidly recognize the pathogenic viral genome via activation of a variety of innate immune signaling pathways which are mediated by PRRs. PRRs detect pathogen-associated molecular patterns (PAMPs) or damage-associated molecular patterns (DAMPs), mainly through the signaling of toll-like-receptors (TLRs), retinoic acid-inducible gene I (RIG-I)-like receptors (RLRs), and nucleotide-binding oligomerization domain (NOD)-like receptors (NLRs) [3,7,14,15]. Consequently, PRR activation drives the transcription of IFN regulatory factors (IRFs) and NF-κB. IRFs further regulate transcriptional factors in the nucleus, which, in turn, stimulate the unique downstream signaling of IFNs and other pro-inflammatory cytokines to induce antiviral response [13,14,15,16].

### 2.1. Virus Recognition by TLRs

As described elsewhere [17], TLRs are type I integral membrane glycoproteins that recognize a variety of respiratory viruses. To date, the expression of 10 TLRs (TLR1-10) has been identified in human respiratory epithelial cells [18,19]. The interaction between TLRs and respiratory viruses trigger the signaling cascades of mitogen-activated protein (MAP) kinases and several transcription factors, including NF-κB, activating transcription factor 2 (ATF2), IRF-3, and IRF-7/9, leading to the secretion of type I (α and β) and type III (λ) IFNs, and pro-inflammatory cytokines (IL-1, IL-6, TNFα) and chemokines (IL-8, MCP-1) [19,20,21]. TLRs contribute to both viral clearance and disease pathogenesis during infection in COPD [22,23,24,25].

TLRs are expressed either on the cell surface (TLR-1, -2, -4, -5, -6, and -10) or in the endosome compartment (TLR-3, -7, -8, and -9). Intracellular TLRs, in particular, TLR3 and TLR7, sense double (ds)- and single-stranded (ss) RNA viruses, including respiratory syncytial virus (RSV), rhinoviruses, influenza viruses, and SARS-CoV-2 [20,21,26]. Apart from TLR3, which signals through TRIF, all other TLRs utilize their cytoplasmic toll-interleukin-1 receptor (TIR) domain to regulate cellular responses via their adaptor protein, MyD88, and kinases to initiate innate inflammatory responses [18,21,26]. In addition, TLR activation upregulates signaling via the reactive oxygen species (ROS)-TNFα-converting enzyme (TACE)-epidermal growth factor receptor (EGFR) pathway, which results in IL-8 and vascular endothelial growth factor (VEGF) production [27]. The cell surface receptors (TLR-2, -4, and -6), however, might also be involved in the recognition of respiratory viruses by the airway epithelium [28,29]. The RSV F and G proteins have been shown to interact with TLR4, TLR2, and TLR6 to modulate the innate immune response [29,30,31]. Most recently, TLR2 has been implicated in sensing the SARS-CoV-2 envelope protein to initiate virus-induced cytokine production, which is correlated to disease severity [28]. Interestingly, cigarette smoke enhanced TLR2 and TLR4 expression in the lung tissue of rodents and humans [25,32,33,34], suggesting a link to increased responses to COVID-19 in COPD airways. In addition, TLR2 and TLR4 may also contribute to COPD inflammation [35].

### 2.2. Virus Recognition by RLRs

RIG-I (encoded by the DDX58 gene), melanoma differentiation-associated gene 5 (MDA5; encoded by the IFIH1 gene), and laboratory of genetics and physiology 2 (LGP2) are RLR family members present in the cytosol that detect intracellular dsRNA and ssRNA of a number of viruses [14,15,16,36]. RLRs bind RNA through a C-terminal helicase domain, which promotes a conformational change that exposes the N-terminal caspase activation and recruitment domain (CARD) [14,15,16,17,18,19,20,36]. In airway epithelial cells, induction of type I and type III interferon antiviral responses have been demonstrated for several respiratory viruses via the interaction of RIG-I/MDA5, CARD, and their adaptor proteins mitochondrial antiviral-signaling protein (MAVS), IFN-β promoter stimulator 1 (IPS-1), leading to activation of downstream NF-κB and IRF-3 pathways [14,15,36,37,38].

On the other hand, the intracellular sensor cGAS (cyclic GMP-AMP (cGAMP) synthase) detects retroviral replication products, dsDNA and RNA/DNA hybrids, to induce the synthesis of cGAMP, which binds and activates STING (stimulator of interferon genes) to regulate type I/III IFN signaling [3,4,13,14,15,16,17,18,19,20]. Moreover, STING and MAVS also regulate downstream multiple kinase signaling cascades, leading to IRF-3 phosphorylation and NF-κB nuclear translocation, and the production of inflammatory cytokines/chemokines [14,15]. Lung tissue from COPD patients shows a constitutive decreased expression of IFN-β, IRF-7, RIG-I, and MDA5, suggesting that this deficiency contributes to increased susceptibility of the COPD epithelium to RNA virus infection [38]. Elevated levels of self-DNA have been linked to cigarette-smoke-induced inflammation in mice, and poor prognosis in COPD [39,40,41]. This cell-free DNA may result from cigarette-smoke-induced oxidative stress, cellular senescence, and apoptosis- or necrosis-associated cell death [39,40]. Self-DNA is also recognized by cGAS-STING DNA virus sensors to regulate type I IFN-dependent lung inflammation in COPD [41].

### 2.3. Virus Recognition by NLRs

NLRs represent another major group of cytoplasmic pathogen recognition receptors. The sensing of DNA and RNA viruses (e.g., RSV, influenza A virus (IAV), measles, encephalomyocarditis virus, hepatitis C virus) via NLRP3 (NLR family pyrin domain containing 3) activation represents a common pathway for viral detection by host cells [42,43,44,45,46,47]. NLRP3 modulates inflammasome-induced inflammation through the activation and secretion of pro-IL-1β and pro-IL-18. The maturation of these cytokines further stimulates the production of caspase-1, IFNs, and other cytokines [44,45,46,47,48]. This is detrimental to the virus, leading to pyroptosis and elimination of virus-infected cells from the host [42,43]. NLRP3 also has a strong link to mitochondrial dysfunction [49,50]. Mitochondria contribute to antiviral responses in cells by facilitating RLR signaling [50]. Elevated NLRP3 has been reported in an in vivo mouse model of COPD exacerbation, and in the lung epithelium of COPD patients during exacerbation [51,52]. The NLRP3 inflammasome induces mitochondrial damage and mitophagy in COPD epithelium, resulting in marked impairment of MAVS-mediated antiviral immunity, thus regulating the production of antiviral type I and type III IFNs, and pro-inflammatory cytokines following viral infection [53,54,55]. Some respiratory viruses, such as rhinoviruses, induce mitochondrial dysfunction to impair the efficiency of host antivirus defense [56,57]. Cigarette-smoke-associated mitochondrial dysfunction and the impaired antiviral response may contribute to the increased susceptibility to respiratory infections in COPD epithelium.

### 2.4. Viral Evasion during Virus Sensing

Respiratory viruses use different strategies to disguise themselves from host-sensing systems to avoid being recognized by the host as dangerous. In order to readily replicate in host cells, rhinoviruses and coronaviruses (CoV, including SARS-CoV-2) modify their recognizable viral nucleic acid products in the cytosol to form ‘replication organelles’ (RO), which can shield from host-sensing signaling cascades [58,59,60,61,62,63,64]. RNA viruses, such as CoVs, rhinoviruses, and RSV, replicate in the cytosol of respiratory epithelial cells, and can form ROs [62]. In the cytosol, RSV induces the formation of ‘inclusion bodies’ that are found associated with cytosolic occluded structures [60]. Non-structural protein (nsp)-3 and nsp4 of CoVs, and the N and P proteins of RSV generate similar intracellular membrane structures inside membrane-bound vesicles or invaginations that are undetectable by host virus sensors, thus promoting productive virus replication machinery [61,62,63].

It is still unclear whether the host innate defense system can also recognize and attack virus replication organelles to restrain virus infection. However, the activation of the type I IFN signaling cascade may induce effectors that compromise the integrity of ROs [62,63]. ROs may be recognized and targeted by GBPs (guanylate-binding proteins), including Mx GTPases, to initiate an antiviral response, and disrupt viral replication [64,65,66,67,68].

Interestingly, unlike other RNA viruses, influenza virus circumvents RIG-I and MDA5 RNA sensors and TLRs by replicating in the nucleus rather than in the cytosol [69]. However, as mentioned above, the host can initiate countermeasures against influenza amplification by GBPs that are localized in the nucleus and the cytosol [69,70].

Some respiratory viruses also avoid host innate immune viral sensing by directly modifying their RNA by adding a cap or a cap mimic-structure to the 5′-end, which is identical to host mRNA [71,72,73,74,75]. Rhinovirus can attach a cap-mimicking peptide, VPg, to its RNA 5′-end to protect it from recognition by innate RNA sensors [71,72,73]. The RNA-dependent RNA polymerase (RdRp) complex of IAV cap-snatches host mRNA from short, 5′-capped transcripts produced by the DNA dependent RNA polymerase II (RNAPII) in the nucleus to prime transcription of viral mRNA [74]. RSV and CoVs utilize the enzymatic function in their polymerase complexes to add cap-structures to their own mRNAs to escape detection by host innate sensors [75,76]. CoVs use the 2′-O-methyltransferase activity of nsp16 to methylate their cap structures to avoid MDA5 and associated innate immune responses [76].

Respiratory viruses also employ endoribonuclease activity to avoid virus sensing. CoVs and influenza encode endonuclease activity to destroy their own RNA, thus preventing viral recognition [77,78]. Likewise, some respiratory viruses induce host shut-off; a process in which viruses halt cellular protein expression, thereby suppressing innate immune responses, and facilitating viral replication and evasion [79,80,81,82]. The NS1 and PA-X proteins of influenza virus play important roles in host shut-off during influenza infection [80]. NS1 inhibits 3′-end cleavage and polyadenylation of cellular mRNAs, preventing host gene expression, including IFN and pro-inflammatory responses [62,80]. The NSP1 protein of CoVs, including SARS-CoV and Middle East respiratory syndrome-related coronavirus (MERS-CoV), mediates host shut-off by binding to cellular transcriptional and translational factors of host mRNA [81]. The 2A protease of rhinoviruses can also cleave host translation initiation factor elF4G to induce host shut-off during infection [82].

Viral infection is associated with the formation of stress granules, an integral part of host stress responses, to store stalled untranslated mRNAs, and coordinate cellular processes during stress, including antiviral responses [83,84,85]. Some RNA viruses, such as RSV, rhinoviruses, influenza viruses, and MERS-CoV, counteract the formation of stress granules to benefit their replication [85,86]. Stress granule factor proteins, such as G3BP1 and G3BP2, have been implicated in the formation of antiviral stress granule formation during RSV, rhinovirus, and SARS-CoV-2 infections [87,88]. Rhinoviruses use their 2A papain-like protease (PLpro) to cleave host proteins in the innate immune response to facilitate invasion [62]. A classic example of this is the RNA-sensing effector, MAVS, which is cleaved by both 2A and 3C proteases of rhinovirus to impede type I IFN signal transduction [62]. Some RNA viruses suppress the host innate immune response by manipulating the ubiquitin system and associated ISG15 protein [62,89,90]. CoV PLpro interact with the deubiquitinating activity of ISG15 and E3 ligase RCHY1 to inhibit type I IFN secretion and apoptosis [91]. In addition, the NS1 protein of influenza and RSV targets TRIM25 to suppress RIG-I ubiquitination, and thus, benefit virus infection [92,93]. Influenza NS1 has been reported to induce the expression of the deubiquitinase A20 to downregulate RIG-I activation [12]. Influenza NS1 was also recently shown to affect the deubiquitinating activity of the MDM2 E3 ligase in a way that supports IAV infection [94].

## 3. Type I and Type III Interferon Signaling Pathways in Airway Epithelium

Interferons are key defense molecules, which control and eliminate viral replication, thus playing a critical role in the host antiviral response. There are three distinct types of interferons, namely type I, type II, and type III interferons [95,96]. Though type II interferon (IFN-γ) is mainly expressed by immune cells, type I and type III interferons are expressed by airway epithelial cells, and will therefore be the focus of the remainder of this review.

### 3.1. Type I Interferon Signaling

**Figure 2:**
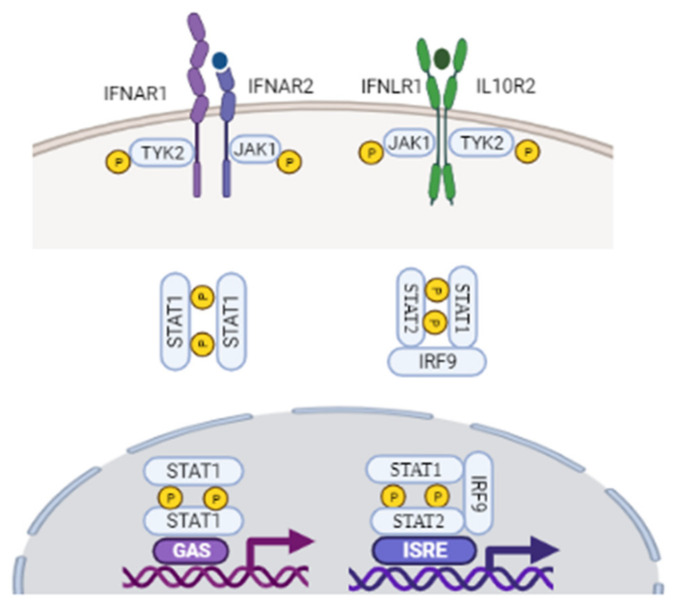
Upon binding to their receptors, IFNs induce the activation of the JAK/STAT signaling pathway. Type I IFNs first bind to the extracellular part of type I IFN heterodimeric receptor complex IFN-α/β R1 and IFN-α/β R2. Receptor engagement subsequently activates IFN-alpha/beta R1 and IFN-α/β R2, resulting in conformational changes in their associated intracellular tyrosine residues Tyk2 and JAK1 protein tyrosine. Both type I and III IFNs use JAK1 for their signaling. Apart from IFNL-R1 receptor, type III IFNs also use IL-10 receptor beta (IL-10R2) receptor complex. Following JAK activation, STAT1/STAT2 are recruited and activated, which leads to their dimerization and binding to IRF9 and ISRE, forming the ISGF3 complex. Upon JAK activation, STAT1 homodimer complex are also formed, which translocate into the nucleus, and drives ISG production. STAT1 homodimers interact with GAS to induce a pro-inflammatory response. GAS, gamma activated sequences; IFN, interferon; IRF9, interferon regulatory factor 9; ISGF3, interferon stimulated gene factor 3; ISRE, interferon-stimulated regulatory element, JAK, Janus kinase; STAT, signal transducer and activator of transcription; TYK2, tyrosine Kinase 2. Figure created with BioRender.com (accessed on 10 October 2021).

Human type I interferons consist of 17 subtypes, including 13 isoforms of IFN-α, along with IFN-β, IFN-ε, IFN-κ, and IFN-ω [18,97,98]. Among these, IFN-α and IFN-β are the most studied, classical, antiviral cytokines. Upon viral infection, however, airway epithelial cells mainly secrete IFN-β protein, and IFN-α is undetectable [99]. Type I IFN production is predominately initiated by IRF-3 and IRF-7 [97,98,100]. It has been well documented that IFN-β is mainly produced from infected cells via IRF-3, and that IFN-β receptor binding induces IRF-7, leading to late-phase IFN-β and IFN-α production [98,101]. As described in Figure 2, type I interferons are translated and secreted from the infected cell to exert their antiviral effect through autocrine and paracrine pathways [97,98]. Type I IFN receptors are found on most cell types, including epithelial cells in the lung, and they signal through canonical and non-canonical pathways to mediate the expression of ISGs [89,97]. As outlined in Figure 2, they first bind to the extracellular part of type I IFN heterodimeric receptor complex IFN-α/β R1 and IFN-α/β R2 on the surface of surrounding cells. Receptor engagement subsequently activates IFN-α/β R1 and IFN-α/β R2, resulting in conformational changes in Tyk2 protein tyrosine kinase for the former, and Janus tyrosine kinase JAK1 for the latter [97,98]. JAKs mediate the phosphorylation and activation of the signal transducer and activator of transcription (STAT) family of proteins through the classical JAK–STAT-signaling pathways [97,98,99,100,101,102].

Upon activation, STAT1 and STAT2, together with IRF-9, regulate the dimerization and the assembly of the IFN-stimulated gene factor 3 (ISGF3), which then translocates to the nucleus to induce transcription of ISGs. JAKs also mediate the phosphorylation of STAT1 and STAT3 homodimer complexes. STAT1 homodimers regulate signaling through gamma-activated sequences (GAS) to induce pro-inflammatory responses [3,4,98,102]. STAT3 homodimers indirectly suppress the transcription of inflammatory genes by activating genes associated with *NF-κB* gene inhibition [97,98], thus limiting the innate pro-inflammatory response. These signaling cascades comprise the canonical type I IFN pathway, which induces the downstream transcription of an array of ISGs to further limit viral infection [97]. The suppressor of cytokine signaling (SOCS) family of proteins inhibit the JAK-STAT signal transduction pathway by forming a negative feedback loop [102,103]. Type I IFNs also signal through the non-canonical pathway to initiate the transcription of ISGs. The main non-canonical pathways used by type I IFNs include the mitogen-activated protein kinase (MAPK) pathway and the phosphoinositide 3-kinase (PI3K)/mammalian target of rapamycin (mTOR) pathways. Other non-canonical pathways include sirtuin (SIRT)-2 and the Schlafen (SLFN) family of proteins [97,98].

Many respiratory viruses, such as RSV and SARS-CoV-2, evade host antiviral responses by restraining the production of type I IFNs [104,105]. The NS1 and NS2 proteins of RSV inhibit the type I IFN host response by interrupting signal transduction and activation of the JAK-STAT pathway [105]. The NSP1, NSP3, ORF3b, ORF6, and N proteins of SARS-CoV are also type I IFNs antagonists [106,107]. Interestingly, ORF6 also suppresses the expression of mRNA export proteins’ Nup98-Rae1 complex by blocking STAT1 nuclear translocation [108]. PLpro inhibits IRF-3 phosphorylation to prevent its homodimerization and nuclear translocation, and antagonizes innate immunity by acting on downstream NF-κB and IFN-β pathways in both SARS-CoV- and SARS-CoV-2-infected cells [109,110]. Some viruses, such as RSV, rhinoviruses, and influenza viruses, induce the expression of *SOCS* genes to inhibit the antiviral effect of ISGs and IFN induction [103,111,112]. Reduced expression of IFN-β and its transcription factor IRF-7 have been demonstrated in the COPD airway epithelium and macrophages compared with non-COPD cells [38].

### 3.2. Type III Interferon Signaling

Type III IFNs (IFN-λ) are a relatively new class of IFNs, which include IFN-λ1/IL-29, IFN-λ2/IL-28A, IFN-λ3/IL-28B, and the most recently discovered, IFN-λ4 [98,113]. *IFN-λ1* gene expression is regulated by both IRF-3 and IRF-7, whereas *IFN-λ2/λ3* genes appear to be induced only by IRF-7 [98,113]. However, influenza virus- and rhinovirus-induced EGFR activation suppressed IRF-1-induced *IFN-λ* production, and increased viral infection [114]. Type III IFNs share similar antiviral pathways with those of type I IFNs, but expression of IFN-λ receptors is largely restricted to epithelial cells, suggesting a frontline role in the antiviral response. The IFN-λ receptor is composed of *IL-10R2* and *IFNLR1* [113,115,116]. As *IL-10R2* is ubiquitously expressed, the expression of *IFNLR1* is restricted to certain cell types, including airway epithelial cells, suggesting that differential transcriptional regulation of the *IFNLR1* gene is associated with the cell-type specificity of IFN-λ.

Despite differential receptor constitution, type III IFNs also act through the canonical signaling pathways, and are recognized by the same PRRs as those used by type I IFNs (Figure 2). Upon virus binding, receptor-ligand complexes trigger JAK1 and Tyk2 phosphorylation and activation, resulting in phosphorylation of STAT1:STAT2 heterodimers. These heterodimers signal through ISGF3, as described in type I IFN pathways, to promote the transcription of downstream ISGs, and induce an antiviral state [115,116,117,118].

### 3.3. ISGs Induce an Antiviral State in Airway Epithelium

IFNs induce a myriad of ISGs, which introduce a second round of autocrine and paracrine signaling in cells. This has the effect of establishing an antiviral state in the host cells to attenuate viral-associated pathogenesis in the infected cells before trigging the adaptive immune response, leading to viral clearance [112,115,116,117,118]. ISGs interact with transcription factors and other signaling mediators to amplify the innate immune response. ISGs also interact with antiviral proteins to disturb virus replication by degrading viral RNA or blocking host cell translation [117,118]. Consequently, ISGs efficiently prevent further spread of the infection in bystander cells by restricting viral transcription and replication [4,118]. ISGs regulate the production of the pro-apoptotic p53 protein and several antiviral proteins, including the GTPase myxovirus resistance 1 (MX1), IFN-inducible double-stranded RNA-dependent protein kinase (PKR), 2′-5′-oligoadenylate synthetase (OAS), IFN-induced transmembrane proteins (IFITMs), apolipoprotein B mRNA-editing enzyme catalytic polypeptide 1 (APOBEC1), the tripartite motif-containing (TRIM) family of molecules, and viperin (virus inhibitory protein, endoplasmic reticulum-associated, interferon-inducible) to subvert viral replication [118,119]. RSV, rhinoviruses, and influenza viruses induce the expression of viperin in airway epithelium to fight against respiratory viral invasion [119,120,121].

Accumulated evidence suggests that many viruses escape host antiviral defenses by preventing ISG secretion, and thus, perturb IFN-α/β and associated signaling pathways. The influenza virus NS1 protein has been shown to inhibit PKR activation, resulting in increased viral replication in the lung [122]. Impaired IFN-λ and IFN-β have been demonstrated in airway epithelial cells of active smokers compared with that of non-smokers [123]. Airway epithelium in asthma and COPD patients have been reported to have a deficient or delayed *IFN-β* and *IFN-λ* response to rhinovirus infection, as well as the secretion of downstream ISGs, including *PKR*, *OAS1*, and *viperin* [9,10]. Elevated IL-17A in the lung tissue of patients with severe COPD correlated with lung function decline, and IL-17A attenuated virus-induced *IFN-λ* expression by enhancing *SOCS1* and *SOCS3* expression to inhibit autocrine signaling loops in human airway epithelial cells [124]. Downregulated *MxA*, *OAS1*, and *viperin* have also been shown in the sputum of severe COPD patients [125]. This defective IFN response in the respiratory tract may contribute to the increased susceptibility to infection resulting in impaired apoptosis and increased virus load in infected cells. Singanayagam and colleagues found that ISG-induced CXCL10 downregulation in immune cells from the lungs of COPD patients was associated with impaired IFN-α, IFN-β, and IFN-λ secretion following rhinovirus infection compared with cells from healthy subjects [9]. Impaired *IFN-β* and *IFN-λ* have also been demonstrated in IAV infected primary bronchial epithelial cells from COPD patients [12,38].

## 4. SARS-CoV-2 Associated IFN Signaling and Implications for COPD

As a global pandemic, SARS-CoV-2 has had a hugely detrimental impact worldwide. SARS-CoV-2 is a non-segmented, positive sense, single-strand RNA virus, which causes both upper and lower respiratory tract infections [126,127]. The majority of patients exhibit mild flu-like upper respiratory symptoms; however, a subset of patients develop severe life-threatening acute respiratory distress syndrome (ARDS) [126,127,128]. Upon infection, individuals with COPD and current smokers are among the high-risk conditions associated with the development of SARS-CoV-2-associated disease and mortality [128,129,130,131]. The infection is initiated by the attachment of the virus (spike) S protein to the host cellular entry receptor angiotensin converting enzyme 2 (ACE2), and other virus entry co-factors, such as transmembrane serine protease 2 (TMPRSS2). Elevated expression of ACE2 has been found in airway epithelium of COPD and smokers [130,131,132]. ACE2 in COPD has also been shown to inversely correlate with lung function [130]. Upregulated ACE and TMPRSS2 expression has been demonstrated on non-ciliated goblet cells (primary target of SARS-CoV-2 infection) in COPD airway epithelium [132]. Increased replication has also been demonstrated in well-differentiated COPD bronchial epithelial cells compared with cells from heathy subjects, suggesting that COPD-associated goblet cell hyperplasia may contribute to the increased susceptibility to SARS-CoV-2-associated severe disease [132]. Most recently, ACE2 has been identified as an ISG, suggesting that SARS-CoV-2 may exploit IFN-driven ACE2 upregulation to enhance infection. [133]. Another study found that SARS-CoV-2 may bind and activate TLR4 to increase ACE2 expression, facilitating entry and virus-induced hyperinflammation, indicating a role of TLR4 activation in SARS-CoV-2 pathogenesis and associated innate immune response [134]. Upregulation of TLR4 has also been implicated in COPD inflammation and exacerbations, and may be a therapeutic target in SARS-CoV-2-induced severe disease [23].

SARS-CoV-2 perturbs host antiviral innate immune responses through multiple elaborate viral inhibitory mechanisms to evade the host immune response, as well as facilitating viral transmission and adaptation to its human host. These strategies include compromising PRR sensing through RIG-I and TLR signaling, antagonizing IFN production, and inhibiting IFN signaling [107,135,136,137].

SARS-CoV-2 infection also induces overt, but delayed, type-I IFN response by inhibiting IFN signaling during the early phase of infection [107,110,138]. Xia and colleagues have demonstrated that the nsp1, nsp6, nsp13, ORF3a, M, and ORF7b proteins of SARS-CoV-2 block STAT1 phosphorylation; whereas nsp6, nsp13, ORF7a, and ORF7b suppress STAT2 phosphorylation, and ORF6 inhibits nuclear translocation of STAT1 [107]. SARS-CoV-2 nsp1, nsp3, nsp12, nsp13, nsp14, orf3, orf6, and M protein have also been shown to inhibit Sendai-virus-induced IFN-β promoter activation, and ORF6 inhibits both type I IFN production and downstream signaling [107,138].

The SARS-CoV-2 PLpro protein exits its deubiquitinating and deISGylating activity by directly removing ubiquitin-like ISG15 modifications from IRF3 and MDA5, and directly cleaving IRF. The PLpro protein can interact with key regulators in the STING, NF-κB, MAPK, and TGF-β pathways, and thereby suppress the innate immune response [110,139,140,141,142]. Therefore, PLpro may also be an important therapeutic target for the treatment of SARS-CoV-2.

In summary, SARS-CoV-2 utilizes mechanisms to circumvent host antiviral immunity, thus facilitating virus replication and spread. Direct IFN-β treatment has shown to effectively block SARS-CoV-2 replication in the early stage of infection [138]. Impaired IFN responses have also been demonstrated in stable COPD airway epithelium, and during COPD exacerbations. Taken together, impaired IFN responses may contribute to the increased susceptibility to SARS-CoV-2-associated severe disease and SARS-CoV-2-induced exacerbations in COPD airway epithelium.

## 5. Therapeutic Implications of IFN Signalling Cascades in COPD Exacerbations: Current Development and Future Perspectives

TLR activation initiates host self-defense mechanisms to fight bacteria or viral pathogens. However, dysregulated TLR2 and TLR4 expression and signaling activation have been demonstrated in cigarette-smoke-induced airway inflammation, and during COPD exacerbations [23,35]. The activation of these receptors initiates downstream MyD88- and NF-κB-dependent signaling cascades, and the associated inflammatory response in the airways [23,35,143]. Inhibition of TLR2 and TLR4 signaling pathways may represent an effective strategy in correcting the excessive inflammatory response in COPD. In response to TLR3 and TLR7/8 stimulation, lung tissue explants from COPD patients display elevated proinflammatory response compared with healthy smokers [144].

In theory, inhibitors or antagonists of TLR signaling that directly block TLR receptor- ligands binding or TLR signaling transduction pathways may represent potential therapeutic targets for treating COPD inflammation. However, this approach is still in its infancy, and to date there are no clinical studies of TLR antagonists in COPD. A number of TLR blockers are currently in Phase II clinical trials for the treatment of other inflammatory conditions, and may offer a promising future perspective for this approach [35,143]

The targeting of mediators in the IFN signaling or even augmentation of IFN signaling may also protect COPD patients from viral-induced exacerbations, thus improving quality of life. In a randomized clinic trial, orally inhaled IFN-β therapy in corticosteroid-resistant asthmatic patients resulted in improved lung function, and a reduced requirement for oral corticosteroids [145] However, there are side effects associated with IFN-β administration which may hamper the clinical application of this approach [146]. In a recent study, Watson et al. reported that exogenous IFN-β treatment of influenza-infected COPD macrophages and airway epithelium had no effect on virus shedding, nor IFN and ISG production [147]. However, giving IFN-β as a prophylactic before infection significantly improved antiviral efficiency in both COPD macrophages and epithelium, indicating that the timing of IFN admission may be critical in clinical trial design. Data from a Phase II randomized trial of on-demand inhaled interferon beta-1a in severe asthmatics subjects with upper respiratory tract infection indicated that inhaled interferon beta-1a treatment did not reduce the severe exacerbation rate, but improved lung function [148].

In conclusion, mediators that regulate IFN antiviral response, and the complex network of IFN signaling transduction, may play an important role in host defense against viral-induced COPD exacerbation. Elucidating the role of IFNs signaling cascades in the pathogenesis of COPD may provide a platform to understand the disease mechanism in this condition, leading to new routes to combat virus-induced exacerbations in COPD.

## Data Availability

Not applicable.
